# Evaluating changes in pulse transit time drop index in patients with obstructive sleep apnea before and during CPAP therapy

**DOI:** 10.1111/crj.13532

**Published:** 2022-08-08

**Authors:** Elham Kalantari, Forough Kalantari, Maryam Edalatifard, Besharat Rahimi

**Affiliations:** ^1^ Department of Pulmonology Isfahan University of Medical Sciences Isfahan Iran; ^2^ Department of Nuclear Medicine, Rasoul Akram Hospital Iran University of Medical Sciences Tehran Iran; ^3^ Advanced Thoracic Research Center Tehran University of Medical Sciences Tehran Iran

**Keywords:** continuous positive airway pressure, CPAP, obstructive sleep apnea, pulse transit time

## Abstract

Airflow limitation in patients with obstructive sleep apnea (OSA) leads to arousal, increased sympathetic nervous system activity, and elevated blood pressure, which causes a decrease in pulse transit time (PTT). The present study aims to evaluate the effect of CPAP therapy on PTT in patients with moderate to severe OSA. This was a cross‐sectional study. Split‐night polysomnography (PSG) study was performed for each participant with apnea‐hypopnea index (AHI) ≥ 15 before and during CPAP therapy. The PTT was calculated as the time interval between the R wave of the electrocardiogram and the following arrival point in fingertip photoplethysmography. PTT drop was defined as a fall in the PTT curve of ≥15 ms lasting at least for 3 s and at most for 30 s. PTT drop index was defined as the number of drops in PTT that occur per hour of sleep. A total of 30 patients were included. PTT significantly increased, and PTT drop index significantly decreased during CPAP therapy (*P* < 0.001). PTT was significantly correlated to sleep efficiency (*r*_s_ = −0.376, *P* = 0.049) and oxygen desaturation index (ODI) (*r*_s_ = −0.428, *P* = 0.018). PTT drop index was strongly correlated to AHI (*r*_s_ = 0.802, *P* < 0.001), respiratory disturbance index (RDI) (*r*_s_ = 0.807, *P* < 0.001), ODI (*r*_s_ = 0.693, *P* < 0.001), arousal index (*r*_s_ = 0.807, *P* < 0.001), and periodic leg movement (PLM) index (*r*_s_ = 0.400, *P* = 0.035). Overall, the findings from this study indicated that the PTT drop index is a non‐invasive and useful marker for evaluating the severity of OSA and the effectiveness of treatment in patients with moderate to severe OSA.

## Graphical abstract

Here, we evaluated changes in pulse transmission time (PTT) drop index before and during CPAP therapy in patients with obstructive sleep apnea. PTT drop index significantly decreased after CPAP therapy. PTT drop index was strongly correlated to AHI, RDI, ODI, arousal index, and PLM index.

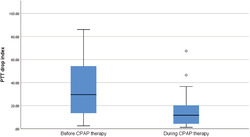

## 1 INTRODUCTION

Obstructive sleep apnea (OSA) is a common sleep‐related breathing disorder characterized by apneas, hypopneas, and respiratory effort‐related arousals (RERAs) caused by the repetitive partial or total collapse of the upper airway during sleep. Polysomnography (PSG) is the gold standard diagnostic test for OSA.^1^ Apnea and hypopneas lead to hypoxemia, which results in chemoreflex activation and overactivity of the sympathetic nervous system.^2^

Pulse transit time (PTT) reflects autonomic imbalances and is a non‐invasive marker of respiratory effort and arousal in patients with OSA.^3^ It represents the time taken for the pulse pressure waveform to travel from the aortic valve to the periphery (usually the finger).^3^ PTT is measured by calculating the time between the R wave of the electrocardiogram (ECG) and the arrival of the pulse wave to distal arterial sites detected by a finger photoplethysmography.^4^ PTT reflects changes in peripheral arterial resistance and intrathoracic pressure and is inversely correlated with blood pressure; with sympathetic activation and increasing blood pressure, the PTT decreases.^5^, ^6^ Moreover, a decrease in intrathoracic pressure during obstructed breathing is associated with a drop in blood pressure, which may be followed by a rise in PTT.^7^, ^8^ Therefore, PTT is an indirect marker of sleep fragmentation and can be used to monitor arousal from sleep due to respiratory events.

Continuous positive airway pressure (CPAP) is the mainstay of therapy for adults with OSA, which reduces the frequency of respiratory events and improves blood pressure.^9^ The present study aimed to evaluate changes in PTT and PTT drop index before and during CPAP.

## 2 METHODS

### 2.1 Patients

Adult patients (age ≥ 18 years) with moderate to severe OSA referred to our sleep clinic from November 2016 to October 2019 were eligible. The diagnosis of OSA was established according to AASM guidelines.^1^ Patients with an apnea‐hypopnea index (AHI) ≥ 15 and < 30 were considered to have moderate OSA, and those with AHI ≥ 30 were considered to have severe OSA. Patients with a history of chronic obstructive pulmonary disease (COPD) or any other chronic respiratory, liver, and kidney diseases were excluded. We also excluded patients with drug‐resistant hypertension, heart failure, and those who receive sedative‐hypnotic drugs.

### 2.2 Polysomnography

A split‐night PSG study was performed for each participant before and during CPAP therapy. PSG data were recorded using the SOMNOscreen PSG system. The standard PSG incorporated electrocardiography, electroencephalography, electrooculography, pulse oximetry, snoring voice recording, electromyography (submental and bilateral anterior tibialis), thoracic and abdominal movements, thermal sensors, and oronasal airflow. Fingertip pulse oximetry and plethysmography were used to monitor oxygen saturation and the termination of the pulse wave. The PSG data were then interpreted automatically and revised manually according to the American Academy of Sleep Medicine criteria.^10^

### 2.3 PTT measurement

The PTT was calculated as the time interval between the R wave of the electrocardiogram (ECG) and the following arrival point in fingertip photoplethysmography.^3^ PTT drop was defined as a fall in the PTT curve of ≥15 ms lasting at least for 3 s and at most for 30 s. PTT drop index was defined as the number of drops in PTT that occur per hour of sleep. PTT events that could be related to an identifiable cause during the recording were also calculated.

1. Respiratory PTT: PTT falls following respiratory events (apnea and hypopnea).
2. Flow limitation PTT: PTT drops following a decrease in upper airflow (which does not meet the criteria for apnea or hypopnea) along with increased respiratory effort.
3. Periodic leg movement PTT: PTT falls following periodic leg movement.
4. Snoring PTT: PTT falls following snoring.
5. Heart rate PTT: PTT falls following an increase in heart rate.
6. Body position PTT: PTT falls following a change in body position.
7. Spontaneous PTT: PTT falls without any recognized physiologic event.

### 2.4 Statistical analysis

Statistical analyses were performed using the SPSS software version 25. Shapiro–Wilk test was used to assess normal distribution. Normally distributed variables were expressed as mean ± standard deviation (SD). Median and interquartile range (IQR) were calculated for non‐normally distributed variables. Spearman's correlation analysis was applied to assess the correlation between PTT and PSG variables. Changes in PTT and PSG variables before and during CPAP therapy were assessed via paired samples *t* tests.

## 3 RESULTS

### 3.1 Patients characteristics

A total of 30 patients with moderate to severe OSA were studied. The mean age was 53.4 ± 11.4 years. Nineteen patients (63.3%) were male. The mean body mass index (BMI) was 33.9 ± 5.3. Obesity (BMI ≥ 30) was detected in 25 patients (83.3%). The AHI ranged from 10.6 to 157.4 before CPAP therapy. Severe OSA was found in 28 patients (93.3%). The mean PTT and PTT drop index before treatment were 309.7 ± 19.6 ms and 34.7 ± 24.4, respectively.

### 3.2 Association between PTT and demographic and polysomnographic findings

**FIGURE 1:**
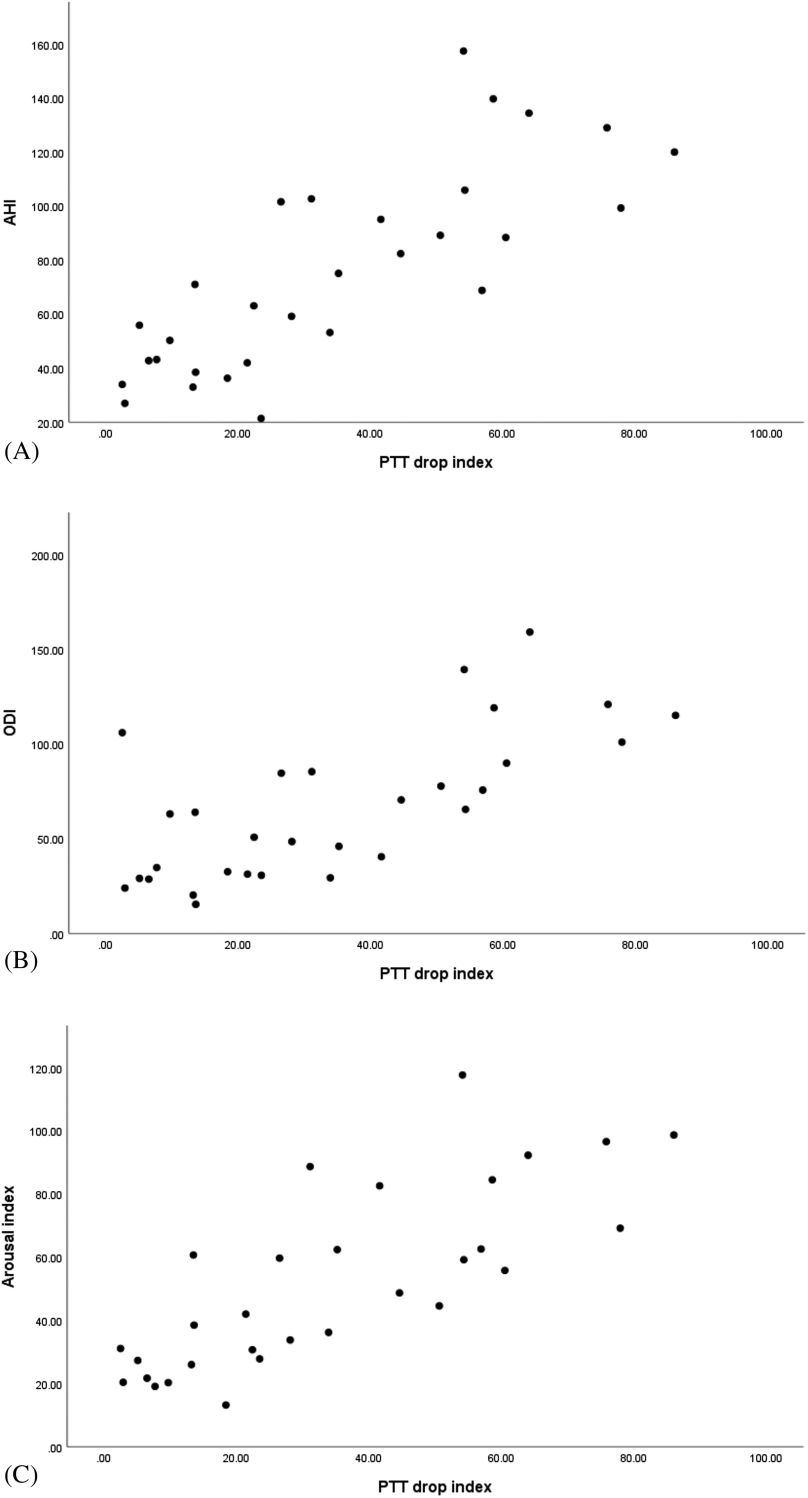
Correlation between PTT drop index and AHI (*r*_s_ = 0.802, *P* < 0.001) (A), ODI (*r*_s_ = 0.693, *P* < 0.001) (B), and arousal index (*r*_s_ = 0.807, *P* < 0.001) (C). AHI, apnea‐hypopnea index; ODI, oxygen desaturation index; PTT, pulse transit time

As is shown in Table 1, PTT was significantly correlated to sleep efficiency and oxygen desaturation index (ODI). No significant association was found between AHI, RDI, and arousal index (*P* > 0.05). PTT drop index was strongly correlated to AHI, respiratory disturbance index (RDI), ODI, arousal index, and periodic leg movement (PLM) index (Figure 1).

**TABLE 1:** Correlation between pulse transit time and demographic and polysomnographic findings

	PTT		PTT drop index	
	*r*_s_	*P* value	*r*_s_	*P* value
Age	0.320	0.085	−0.175	0.354
BMI	−0.089	0.642	0.092	0.628
AHI	−0.208	0.271	0.802	<0.001
RDI	−0.213	0.259	0.807	<0.001
TST	−0.146	0.458	−0.195	0.321
Sleep efficiency	−0.376	0.049	−0.110	0.577
Sleep latency	0.181	0.357	−0.042	0.831
REM	0.151	0.444	−0.364	0.057
ODI	−0.428	0.018	0.693	<0.001
Average SPO2	0.354	0.055	−0.342	0.065
Arousal index	−0.186	0.325	0.807	<0.001
PLM index	0.355	0.064	0.400	0.035

Abbreviations: AHI: apnea‐hypopnea index; BMI: body mass index; ODI: oxygen desaturation index; PLM: periodic limb movement; RDI: respiratory disturbance index; TST: total sleep time.

### 3.3 Changes in PTT and polysomnographic parameters after CPAP therapy

**FIGURE 2:**
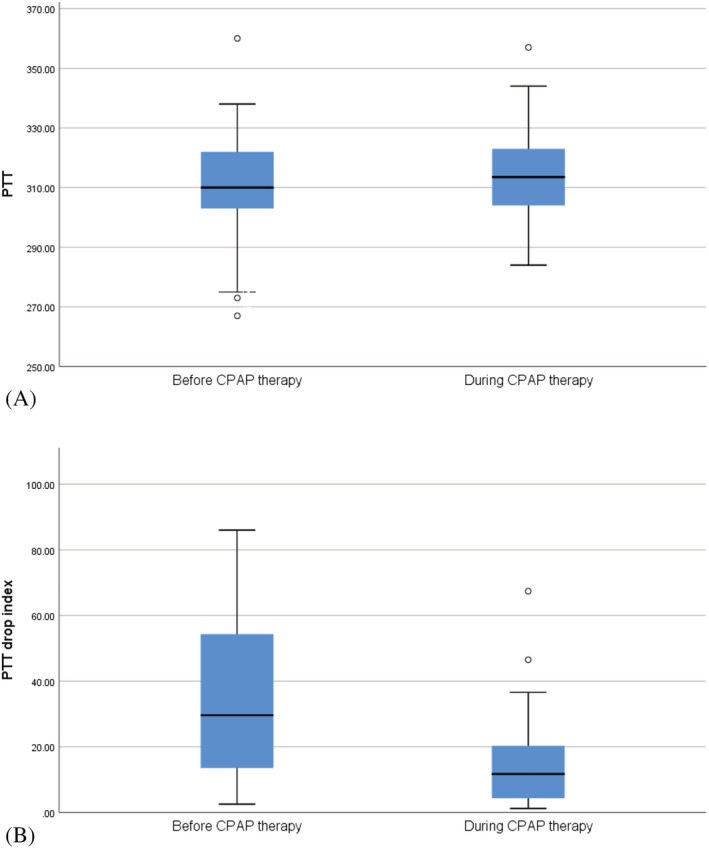
Changes in PTT (A) and PTT drop index (B) before and during CPAP therapy. Error bars are the 95% confidence interval, the bottom and top of the box are the 25th and 75th percentiles, the line inside the box is the 50th percentile, and outliers are shown as open circles. CPAP, continuous positive airway pressure; PTT, pulse transit time

Changes in PTT and other polysomnographic parameters before and after initiation of CPAP therapy are summarized in Table 2. PTT significantly increased, and the PTT drop index significantly decreased after CPAP therapy (*P* < 0.001) (Figure 2). Among PTT drop index parameters, a significant decrease in respiratory, PLM, and snore indexes was found (Table 2). AHI, RDI, sleep efficiency, sleep latency, REM, ODI, average SPO2, arousal, and PLM index improved significantly after CPAP therapy (Table 2).

**TABLE 2:** PTT and other polysomnographic parameters before and after CPAP therapy

	Before CPAP	After CPAP	P‐value
PTT, ms [mean ± SD]	309.7 ± 19.6	315.0 ± 16.3	<0.001
PTT drop index [median (IQR)]	29.6 (13.4–55.0)	11.7 (4.2–20.6)	<0.001
Respiratory PTT [median (IQR)]	8.7 (1.4–34.3)	1.4 (0.0–4.8)	<0.001
Flow limitation PTT [median (IQR)]	0.0 (0.0–0.0)	0.0 (0.0–0.0)	0.593
PLM PTT [median (IQR)]	0.3 (0.0–3.5)	0.0 (0.0–0.6)	0.009
Snore PTT [median (IQR)]	3.7 (1.5–5.5)	1.1 (0.3–4.4)	0.014
Heart rate PTT [median (IQR)]	0.0 (0.0–0.0)	0.0 (0.0–0.0)	1.000
Body position PTT [median (IQR)]	0.0 (0.0–0.0)	0.0 (0.0–0.0)	0.317
Spontaneous PTT [median (IQR)]	4.9 (1.9–11.6)	4.8 (1.7–10.9)	0.888
AHI [mean ± SD]	75.3 ± 37.0	28.1 ± 13.4	0.008
RDI [mean ± SD]	76.1 ± 36.4	29.4 ± 13.4	<0.001
TST, min [mean ± SD]	143.6 ± 49.8	175.3 ± 55.4	0.094
Sleep efficiency [median (IQR)]	78.6 (66.2–87.3)	87.2 (78.8–92.3)	0.006
Sleep latency, [median (IQR)]	8.9 (6.1–22.9)	6.6 (3.8–10.1)	0.030
REM, % of TST [median (IQR)]	0.1 (0.0–8.7)	22.6 (12.5–31.9)	<0.001
ODI [mean ± SD]	66.6 ± 38.6	18.1 ± 3.3	<0.001
Average SPO2 [median (IQR)]	90.5 (88.8–92.3)	92.5 (91.5–94.3)	<0.001
Arousal index [median (IQR)]	46.7 (27.7–72.6)	19.5 (15.7–25.1)	<0.001
PLM index [median (IQR)]	12.5 (1.9–32.5)	4.0 (1.2–8.6)	0.005

Abbreviations: AHI, apnea‐hypopnea index; IQR, interquartile range; ODI, oxygen desaturation index; PLM, periodic limb movement; RDI, respiratory disturbance index; TST, total sleep time.

## 4 DISCUSSION

Obstruction of the upper respiratory tract during sleep in patients with OSA leads to arousal, increased sympathetic nervous system activity, and increased blood pressure, followed by a PTT drop. Therefore, measurements of the PTT drop index can, directly and indirectly, reflect the severity of OSA. In the present study, we observed a strong direct correlation between the PTT drop index and AHI, RDI, ODI, and arousal index, which may confirm this claim. We also monitored changes in PTT and PSG parameters before and after initiation of CPAP therapy. PTT [ms] increased significantly, and the PTT drop index decreased significantly following CPAP therapy. Among PSG parameters, AHI, RDI, sleep efficiency, sleep latency, REM, ODI, average SPO2, and arousal index improved significantly after CPAP therapy.

Although associations between PTT and PSG parameters were evaluated in some previous studies,^5^, ^8^, ^11^, ^12^ only a few had investigated the changes in PTT after positive airway pressure therapy.^13^, ^14^ In a similar study by Schwartz et al., PTT drop index changes before and after CPAP therapy were evaluated.^13^ The authors observed a significant decrease in RDI, ODI, arousal index, as well as PTT drop index just after CPAP therapy.^13^

Investigating identifiable causes of PTT drop before and after CPAP is a unique feature of the present study. Not surprisingly, the majority of PTT drops occurred due to respiratory events, which resolved significantly after CPAP therapy. These findings may indicate that by preventing airflow obstruction, CPAP subsides respiratory events and autonomic overactivity, leading to a lower rate of PTT drops during sleep.

PLM PTT and snore PTT indexes also improved significantly after CPAP therapy. PLM is characterized by repetitive stereotypical movements of the lower extremities during sleep.^15^ It was shown that PLM is associated with increased heart rate and blood pressure as well as EEG arousals and was remarked as a risk factor for nocturnal arrhythmia and hypertension in patients with sleep‐related breathing disorders.^16^, ^17^, ^18^ We showed that the PTT drop index is significantly correlated to the PLM index, and we observed that the PLM index decreased significantly after CPAP therapy. In contrast to our findings, a significant increase in the PLM index was reported after CPAP therapy in some previous studies, especially in patients with severe OSA.^19^, ^20^ To our knowledge, no previous study has evaluated changes in the PTT drop index related to PLM before and after CPAP therapy.

Snoring is a sound produced by the vibration of the upper airway's soft tissues during sleep and indicates increased upper airway resistance and pharyngeal collapsibility. Snoring is a common symptom among OSA patients and usually is associated with excessive daytime sleepiness and an increased risk of cardiovascular diseases.^21^, ^22^, ^23^ A limited number of previous studies have shown changes in the autonomic system following snoring in patients with OSA and those with primary snoring.^24^, ^25^, ^26^ A recently published study has demonstrated that snoring in pregnant women is associated with a significant PTT drop.^27^

Spontaneous PTT was another important determinant of the PTT drop index, which did not change significantly following CPAP therapy. This finding may indicate that PTT drop may occur even without any recordable physiological stimuli during sleep. Spontaneous surges in blood pressure that normally occurs during sleep and/or spontaneous arousals might be responsible for these spontaneous PTT drops.^28^, ^29^ According to current evidence, spontaneous arousals and autonomic activations during sleep increase with age, and AHI has no major impact on that.^29^, ^30^ This might justify no change in spontaneous PTT before and after CPAP therapy.

The findings from the current study should be interpreted cautiously due to some major limitations. Small sample size, single‐night study, and lack of follow‐up are the most important limitations of this study. Since the PTT drop index reflects changes in blood pressure and autonomic nervous system, which are among the main risk factors of cardiovascular disease and mortality in OSA patients, the long‐term monitor of PTT drop index changes in OSA patients receiving CPAP therapy might be of great importance. Furthermore, due to the lack of a healthy control group, the importance of each subindex of the PTT drop index is undetermined.

Here, we showed that the PTT drop index is strongly correlated to AHI, RDI, ODI, and arousal index, which may confirm the utility of the PTT drop index as a non‐invasive marker of OSA severity. We also demonstrated that CPAP therapy significantly improved PTT drop index and some PSG parameters, including AHI, RDI, sleep efficiency, sleep latency, REM, ODI, average SPO2, PLM index, and arousal index. Among subindexes of PTT drop index, respiratory, PLM, and snore PTT reduced significantly following CPAP therapy, but no change in spontaneous PTT was detected.

## CONFLICT OF INTEREST

None of the authors has any conflict of interest to disclose.

## ETHICS STATEMENT

All procedures performed in studies involving human participants were in accordance with the ethical standards of the institutional and/or national research committee and with the 1964 Helsinki declaration and its later amendments or comparable ethical standards. The protocol of this study was reviewed and approved by the Ethics Committee of Tehran University of Medical Sciences. Written informed consent was obtained from all patients included in the study.

## AUTHOR CONTRIBUTIONS

EK contributed to conducting the research, interpreting data, and revising the manuscript, FK contributed to conducting the research and revising the manuscript, ME contributed to the ideas of this study and revising the manuscript, and BR contributed to the ideas of this study, conducting the research, interpreting data and revising the manuscript.

## Data Availability

Data from this trial are available upon reasonable request and approval by the corresponding author.

## References

[1] Kapur VK , Auckley DH , Chowdhuri S , et al. Clinical practice guideline for diagnostic testing for adult obstructive sleep apnea: an American Academy of Sleep Medicine clinical practice guideline. J Clin Sleep Med. 2017;13(3):479‐504. doi:10.5664/jcsm.6506 28162150 PMC5337595

[2] Lombardi C , Pengo MF , Parati G . Obstructive sleep apnea syndrome and autonomic dysfunction. Auton Neurosci. 2019;221:102563. doi:10.1016/j.autneu.2019.102563 31445406

[3] Smith RP , Argod J , Pépin J‐L , Lévy PA . Pulse transit time: an appraisal of potential clinical applications. Thorax. 1999;54(5):452‐457. doi:10.1136/thx.54.5.452 10212114 PMC1763783

[4] Naschitz JE , Bezobchuk S , Mussafia‐Priselac R , et al. Pulse transit time by R‐wave‐gated infrared photoplethysmography: review of the literature and personal experience. J Clin Monit Comput. 2004;18(5–6):333‐342. doi:10.1007/s10877-005-4300-z 15957624

[5] Pitson D , Chhina N , Knijn S , Van Herwaaden M , Stradling J . Changes in pulse transit time and pulse rate as markers of arousal from sleep in normal subjects. Clin Sci. 1994;87(2):269‐273. doi:10.1042/cs0870269 7924174

[6] Pépin JL , Tamisier R , Borel JC , Baguet JP , Lévy P . A critical review of peripheral arterial tone and pulse transit time as indirect diagnostic methods for detecting sleep disordered breathing and characterizing sleep structure. Curr Opin Pulm Med. 2009;15(6):550‐558. doi:10.1097/MCP.0b013e3283318585 19724229

[7] Lea S , Ali N , Goldman M , Loh L , Fleetham J , Stradling J . Systolic blood pressure swings reflect inspiratory effort during simulated obstructive sleep apnoea. Sleep. 1990;90:178‐181.

[8] Pitson D , Sandell A , Van den Hout R . Use of pulse transit time as a measure of inspiratory effort in patients with obstructive sleep apnoea. Eur Respir J. 1995;8(10):1669‐1674. doi:10.1183/09031936.95.08101669 8586120

[9] Giles TL , Lasserson TJ , Smith BJ , White J , Wright J , Cates CJ . Continuous positive airways pressure for obstructive sleep apnoea in adults. Cochrane Database Syst Rev. 2006;1:Cd001106. 10.1002/14651858.CD001106.pub2 16437429

[10] Berry RB , Budhiraja R , Gottlieb DJ , et al. Rules for scoring respiratory events in sleep: update of the 2007 AASM Manual for the Scoring of Sleep and Associated Events. Deliberations of the Sleep Apnea Definitions Task Force of the American Academy of Sleep Medicine. J Clin Sleep Med. 2012;8(5):597‐619. doi:10.5664/jcsm.2172 23066376 PMC3459210

[11] Chakrabarti B , Emegbo S , Craig S , Duffy N , O'Reilly J . Pulse transit time changes in subjects exhibiting sleep disordered breathing. Respir Med. 2017;122:18‐22. doi:10.1016/j.rmed.2016.11.014 27993286

[12] Katz ES , Lutz J , Black C , Marcus CL . Pulse transit time as a measure of arousal and respiratory effort in children with sleep‐disordered breathing. Pediatr. Res. 2003;53(4):580‐588. doi:10.1203/01.PDR.0000057206.14698.47 12612196

[13] Schwartz DJ . The pulse transit time arousal index in obstructive sleep apnea before and after CPAP. Sleep Med. 2005;6(3):199‐203. doi:10.1016/j.sleep.2004.12.009 15854849

[14] Contal O , Carnevale C , Borel JC , et al. Pulse transit time as a measure of respiratory effort under noninvasive ventilation. Eur Respir J. 2013;41(2):346‐353. doi:10.1183/09031936.00193911 22523360

[15] American Sleep Disorders Association . Recording and scoring leg movements. The Atlas Task Force. Sleep. 1993;16(8):748‐759. 8165390

[16] Siddiqui F , Strus J , Ming X , Lee IA , Chokroverty S , Walters AS . Rise of blood pressure with periodic limb movements in sleep and wakefulness. Clin Neurophysiol. 2007;118(9):1923‐1930. doi:10.1016/j.clinph.2007.05.006 17588809

[17] Pennestri MH , Montplaisir J , Colombo R , Lavigne G , Lanfranchi PA . Nocturnal blood pressure changes in patients with restless legs syndrome. Neurology. 2007;68(15):1213‐1218. doi:10.1212/01.wnl.0000259036.89411.52 17420405

[18] Sieminski M , Pyrzowski J , Partinen M . Periodic limb movements in sleep are followed by increases in EEG activity, blood pressure, and heart rate during sleep. Sleep Breath. 2017;21(2):497‐503. doi:10.1007/s11325-017-1476-7 28190164 PMC5399045

[19] Baran AS , Richert AC , Douglass AB , May W , Ansarin K . Change in periodic limb movement index during treatment of obstructive sleep apnea with continuous positive airway pressure. Sleep. 2003;26(6):717‐720. doi:10.1093/sleep/26.6.717 14572125

[20] Fry JM , DiPhillipo MA , Pressman MR . Periodic leg movements in sleep following treatment of obstructive sleep apnea with nasal continuous positive airway pressure. Chest. 1989;96(1):89‐91. doi:10.1378/chest.96.1.89 2661161

[21] Gottlieb DJ , Yao Q , Redline S , Ali T , Mahowald MW . Does snoring predict sleepiness independently of apnea and hypopnea frequency? Am J Respir Crit Care Med. 2000;162(4):1512‐1517. doi:10.1164/ajrccm.162.4.9911073 11029370

[22] Koskenvuo M , Kaprio J , Telakivi T , Partinen M , Heikkila K , Sarna S . Snoring as a risk factor for ischaemic heart disease and stroke in men. Br Med J (Clin Res Ed). 1987;294(6563):16‐19. doi:10.1136/bmj.294.6563.16 PMC1245038 3101779

[23] Hu FB , Willett WC , Manson JE , et al. Snoring and risk of cardiovascular disease in women. J. Am. Coll. Cardiol. 2000;35(2):308‐313. doi:10.1016/S0735-1097(99)00540-9 10676674

[24] Schobel C , Fietze I , Glos M , et al. Nocturnal snoring decreases daytime baroreceptor sensitivity. Respir Med. 2014;108(7):1049‐1055. doi:10.1016/j.rmed.2014.03.012 24735916

[25] Gates GJ , Mateika SE , Mateika JH . Heart rate variability in non‐apneic snorers and controls before and after continuous positive airway pressure. BMC Pulm Med. 2005;5(1):9. doi:10.1186/1471-2466-5-9 16048652 PMC1208915

[26] Jennum P , Schultz‐Larsen K , Christensen N . Snoring, sympathetic activity and cardiovascular risk factors in a 70 year old population. Eur J Epidemiol. 1993;9(5):477‐482. doi:10.1007/BF00209524 8307131

[27] Link BN , Eid C , Bublitz MH , et al. Pulse transit time in pregnancy: a new way to diagnose and classify sleep disordered breathing? Sleep. 2019;42(5). doi:10.1093/sleep/zsz022 PMC6519909 30753641

[28] Chouchou F , Sforza E , Celle S , et al. Pulse transit time in screening sleep disordered breathing in an elderly population: the PROOF‐SYNAPSE study. Sleep. 2011;34(8):1051‐1059. doi:10.5665/SLEEP.1160 21804667 PMC3138160

[29] Bradley J , Galland BC , Bakker JP , et al. Pulse transit time and assessment of childhood sleep disordered breathing. Arch Otolaryngol‐‐Jead Neck Surg. 2012;138(4):398‐403. 10.1001/archoto.2012.86 22508624

[30] Chen H‐C , Lin C‐M , Lee M‐B , Chou P . The relationship between pre‐sleep arousal and spontaneous arousals from sleep in subjects referred for diagnostic polysomnograms. J Chin Med Assoc. 2011;74(2):81‐86. doi:10.1016/j.jcma.2011.01.016 21354085

